# Primary Angiitis of the Central Nervous System: A Report of Three Cases from a Single Colombian Center

**DOI:** 10.1155/2013/940438

**Published:** 2013-05-08

**Authors:** Nicolás Coronel-Restrepo, Fabio Bonilla-Abadía, Omar A. Cortes, Jorge H. Izquierdo, Alberto M. Shinchi, Juan C. Bravo, Gabriel J. Tobón, Carlos A. Cañas

**Affiliations:** ^1^Internal Medicine Unit, Fundación Valle del Lili, CES University, Cali, Colombia; ^2^Rheumatology Unit, Fundación Valle del Lili, ICESI University, Colombia; ^3^Clinical Neurology Unit, Fundación Valle del Lili, Cali, Colombia; ^4^Pathology Unit, Fundación Valle del Lili, Cali, Colombia

## Abstract

The primary angiitis of the central nervous system (PACNS) is an entity with a very low incidence and prevalence. It is not clear why the inflammatory process of this entity is limited to the cerebral vasculature without systemic manifestations. Its clinical manifestations are very heterogeneous and make clinical diagnosis difficult. In most cases, a brain biopsy is required. Only the clinical suspicion and the ability to recognize the possible clinical and imagenological patterns of presentation make an accurate diagnosis possible. The vast majority of the treatment recommendations are given by series of case reports. The following paper described the clinical, imagenological, and histopathological characteristics of three Colombian patients with PACNS. The strategic therapeutic used in shown.

## 1. Introduction

The primary angiitis of the central nervous system (PACNS) is an uncommon disorder of unknown cause that is restricted to the brain and spinal cord, leading to inflammation and destruction of vessels at this level, without evidence of vasculitis outside the central nervous system. An extremely low annual incidence of 2.4 cases per million patient/years is reported [1]. It was considered a distinct clinical entity in 1959 by Cravioto and Feigin [2]. The diagnosis remains a challenge, since there are no universally accepted diagnostic criteria and imaging findings may not be specific. Different types of clinical presentation have been described, which makes it even more difficult to identify. Unlike systemic vasculitis, this entity lacks positive autoantibodies. We described the clinical, imagenological, and histopathological findings in three patients with PANCS treated in a Colombian tertiary-referral hospital.

## 2. Case Report 1

**Figure 1:**
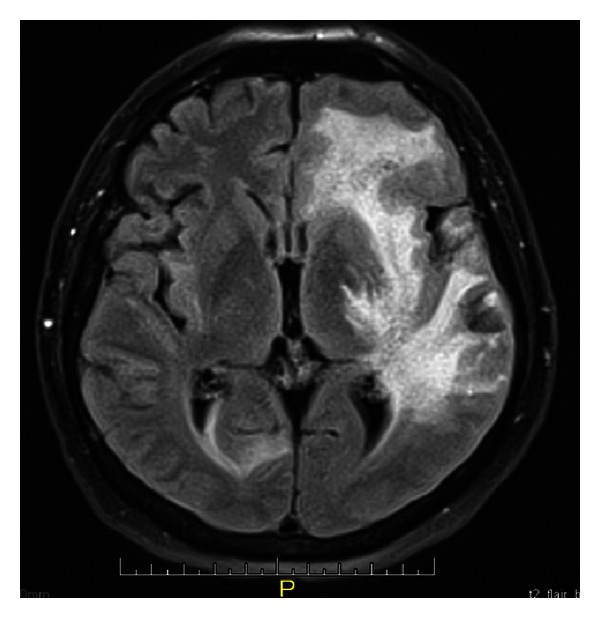
Brain RMI revealed a suggestive image of brain mass at the left parietal lobe, with bilateral bleeding areas predominantly in the left brain.

**Figure 2:**
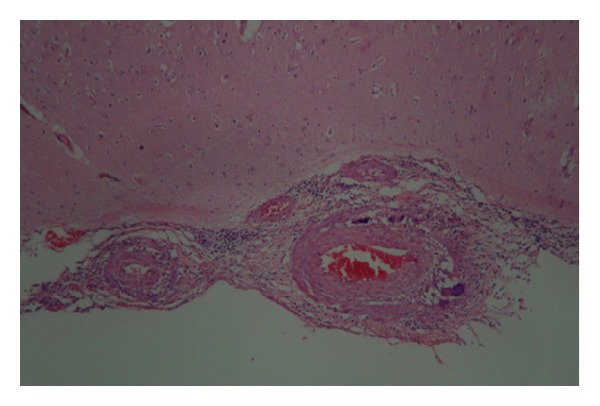
Compromised arterial wall by an inflammatory infiltrate of lymphocytes and multinucleated giant cells. (H & E 10X.)

**Figure 3:**
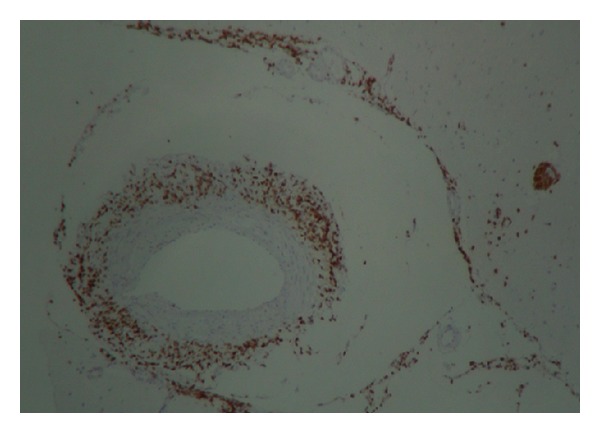
Infiltration of the blood vessel wall by polyclonal T lymphocytes. (Immunohistochemical for CD3 marker.)

A 54-year-old man without any relevant previous history presented with seven months of progressive headache and episodic deficit of memory. Few days before admission he showed abnormal speech, periods of disorientation, right hemiparesis and unstable gait. On admission, his Glasgow Coma Scale score was 13 with slight disorientation, right hemiparesis, and anomia, and motor aphasia. Brain magnetic resonance imaging (MRI) revealed a suggestive image of brain mass at the left parietal lobe, concerning mainly periventricular white matter, semioval centers, with right temporal involvement, ipsilateral occipital, left cerebellar parenchymal, and leptomeningeal enhancement, with bilateral bleeding areas predominantly in the left brain (Figure 1). MRI angiography reported multiple segmental areas of thinning at middle cerebral artery compatible with a vasculitis, which are also present in the anterior cerebral artery. Brain biopsy was performed whose findings were consistent with PACNS and secondary cerebral infarction. There was no evidence of granulomas (Figures 2 and 3). Laboratory examination demonstrated carcinoembryonic antigen, syphilis serology, and HIV test negative; IgG and IgM anticardiolipin, p and c antineutrophil cytoplasmic antibodies (ANCAS), antinuclear antibodies (ANAS), anti-La, anti-Ro, anti-Sm, anti-RNP and anti-DNA antibodies were negative. C3: 148 mg/dL (90–180), C4: 39.5 mg/dL (10–40); CRP: 1.91 mg/dL and ESR: 2 mm/h (2–20). Chest X-ray, ultrasonography and computed tomography of the abdomen were normal. The patient was treated with bolus of methylprednisolone (1 gr IV each day per three days), plus oral cyclophosphamide 100 mg/day, ASA 100 mg/day and phenytoin 300 mg/day. Physical therapy was also indicated. One month later the patient was evaluated in rheumatology and neurology service presenting adequate recovery, with increase in strength of lower right limb.

## 3. Case Report 2

**Figure 4:**
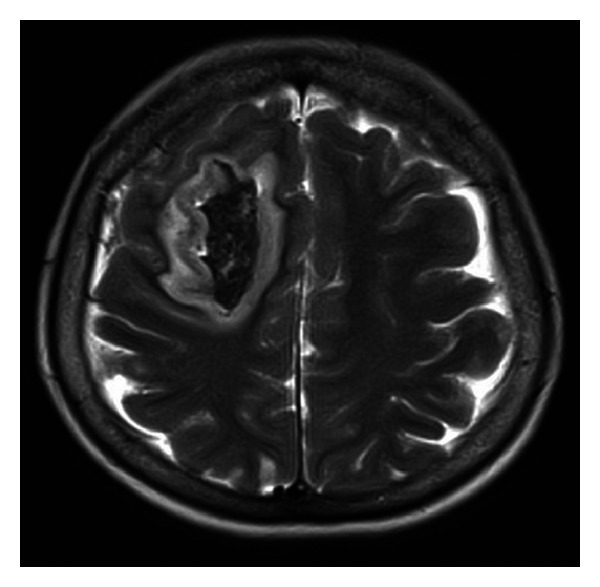
Brain MRI was performed with evidence of intracerebral hematoma with right frontal brain edema.

A 55-year-old woman was admitted to the emergency department displaying clinical evolution of two days consisting of sudden and severe headache associated with syncope and full recovery of symptoms, with a second episode 24 hours later, with left hemiparesis, aphasia, and stupor. There was no history of pathological antecedents of significance. Initially a computerized axial tomography was performed with evidence of a right frontal hematoma and vasogenic edema without deviation from the midline. An impregnation with phenytoin was made. On admission, the patient was tending to sleepiness, with opening ocular to the call and normal ocular movements. She repeated words (transcortical motor aphasia) and showed left hemiparesis with Babinski, without neck stiffness. She was transferred to ICU for monitoring and medical management. A cerebral angiography was performed with evidence of a normal vertebrobasilar system, images of cerebral vasculitis in branches of anterior, middle and posterior right carotid system with a small aneurysm, and infundibular dilation of left posterior communicating artery, without evidence of rupture. Presents image compatible with vasculitis in frontopolar artery. There was no evidence of brain aneurysm or arteriovenous malformation. A PACNS was suspected. Autoimmunity studies were negative and an echocardiogram was normal. Brain MRI was performed with evidence of intracerebral hematoma with right frontal brain edema, which extends into the III and IV ventricles as well as the lateral ventricles (Figure 4). Bifrontal subarachnoid hemorrhage and acute ischemic left parietal and right cerebellum events were reported. Brain biopsy was performed evidencing perivascular lymphocytic infiltrate in meninges without evidence of granulomas. Bolus of methylprednisolone (1 gr each day per three days) and cyclophosphamide (1 gr IV monthly per six doses) was initiated. The patient was discharged with prednisolone 1 mg/kg/day and phenytoin 300 mg/day orally with slow improvement and progressive recovery of his motor functions and language. Currently she is under monthly monitoring, receiving cyclophosphamide and prednisolone orally with dose tapering.

## 4. Case Report 3

A 35-years-old man was admitted to hospital by progressive memory impairment, left hemiparesis, and language disorder. His medical record was relevant to arterial hypertension, left total hip replacement for avascular necrosis, and chronic convulsive syndrome. There was no history of substance abuse. His drug history showed the use of enalapril 20 mg twice a day, Tegretol 1800 mg/day, and phenobarbital 150 mg/day. A control brain MRI showed the presence of hyperintense lesions in the left frontal lobe and paraventricular region with a mass effect. According to these findings and the patient's poor clinical progression we decided to perform brain biopsy documenting necrotizing granulomatous vasculitis. Autoimmune tests such as rheumatoid factor, IgG and IgM anticardiolipin, ANCAS, ANAS, anti-La, anti-Ro, anti-Sm, anti-RNPs and anti-DNA antibodies were negative. CRP: 3.2 mg/dL and ESR: 21 mm/h (2–20). Other autoimmune, infectious, and malignant diseases were discarded. Treatment with cyclophosphamide (1 gr IV single dose) and metilprednisolone (1 gr/day per 3 days) was started continuing prednisone 50 mg/day with gradual decrease to 12,5 mg/day at the sixth month. The patient returned to our service two years after presenting aggressive behavior, mutism, sometimes unmotivated laughter, incontinence, and progressive neurological deterioration, and therefore he was hospitalized. A new brain MRI showed two frontoparietal and left cerebellar active inflammatory lesions compatible with progression of the vasculitis and treatment with metilprednisolone (2 gr/kg/day per three days) was decided to start. However, the patient presented poor response with impairment of the condition, and in this sense rituximab was initiated (2 doses of 1 gr separated by two weeks) but the patient presented with further deterioration and died. An autopsy was not performed.

## 5. Discussion

PACNS is an entity poorly understood and significant challenges remain for diagnosis and treatment [2]. However, there has been an increased recognition with the diagnostic criteria proposed by Calabrese and Mallek and the number of reported cases have risen substantially. The general advances in diagnosis of these neurological disorders have led to an aggressive diagnostic approach and enriched clinical and pathological descriptions [3, 4].

Histologically, the inflammatory process commonly is lymphocytic and affects the medium-sized and small arteries and arterioles of the meninges and cortex of the brain and only rarely the veins and venules. The classic findings of segmental granulomatous vasculitis with multinucleated giant cells occur in less than 50% of patients. Necrotizing vasculitis occurs in 25% of patients. The presence of intimal fibrosis usually signifies healed lesions [5].

The clinical, imaging, and angiography spectra can be very broad. The median age of onset is 50 years, but may affect patients of all ages. The neurological manifestations are diverse, ranging from hyperacute to chronic and insidious. Generally PACNS consist of headache, altered cognition, focal weakness, or stroke occurring in 30–50% of patients, usually affecting many different vessels [4].

Different patterns and outcomes of the disease hve been described, like fulminant-disease onset, spinal involvement [6], prominent leptomeningeal enhancement, and negative cerebral angiography, suggesting that medium-sized vessels are affected. CSF examination reveals evidence for aseptic meningitis in over 90% of patients [7]. Another presentation was described by Molloy et al. [5] characterized by a mass-lesion, making the pathological confirmation essential for diagnosis. Sometimes aggressive immune suppressive treatment could obviate the need for excision [8]. At last, there is a group of vasculitis that is characterized by abnormal cerebral angiography and CSF examination but usually a normal brain in the biopsy. 

The most important differential diagnosis is the systemic vasculitis, which is characterized by the presence of constitutional symptoms and serological markers indicating systemic inflammation [9]. The hallmark of the PACNS is that inflammatory process is limited to the CNS. Diseases that can mimic PACNS are the reversible cerebral vasoconstriction syndrome, premature intracranial atherosclerosis, fibromuscular dysplasia, moyamoya disease, brain neoplasms, genetic conditions (e.g., CADASIL), posterior reversible encephalopathy syndrome (PRES), chronic hypertension (microvascular cerebral ischaemia), demyelinating diseases, and others with multifocal cerebral thromboembolism. It is important to notice that infections (i.e., *Varicella zoster* virus, HIV, and hepatitis C virus) have been associated with cerebral angiographic abnormalities and should be excluded with serological tests [10, 11]. Tuberculosis is another important mimic of PACNS.

No single laboratory test has sufficient sensitivity or specificity to establish the diagnosis, and high clinical suspicion is necessary. Brain biopsy is required to confirm the diagnosis and exclude other causes. Based on published studies, the estimated sensitivity of cerebral angiography for detection of vasculitis is between 27% and 90% and for brain biopsy between 36% and 83% [3, 4]. MRI is the main neuroradiographical modality for the workup of patients with suspected PACNS, and generally, the combination of normal findings on MRI and normal CSF analysis has a high negative predictive value for the diagnosis of PACNS [8].

A common pitfall in the workup diagnosis is to start immunosuppressive treatment without establishment of diagnosis or exclusion of other diseases. Unfortunately no randomised studies of PACNS have been done, and thus all information on treatment is based on retrospective observational data and clinical experience. The efficacy of most immunosuppressive agents such as azathioprine, methotrexate, and rituximab that are used in systemic vasculitis is still unknown or remains elusive in PACNS [9]. The vast majority of case series suggest a high degree of good outcomes when patients are treated with glucocorticoids or glucocorticoids and cyclophosphamide. However, there are some reports using a therapy combining mycophenolate mofetil and steroids allowing control of the disease with disappearance of the neurological abnormalities, restoration of normal daily activities and dramatic improvement in brain MRI abnormalities [12].

The majority of PANCS reports are from Europe and North America [13]. In Latin America there are no reports of PACNS, and we do not really know the incidence and prevalence of this entity in Colombia. Takayasu arteritis was the most frequently reported form of primary vasculitis overall, from Colombia [13], Brazil, and Mexico [14]. Even though we think there is some experience with PACNS in Latin America, it has not been published.

In our experience since 2001 we have reported the identification and management of these three cases of PACNS. All the three cases were documented and studied completely. This may be the first series of PACNS reported in Colombia. Importantly to know the clinical heterogeneous presentation of this condition when we handled this group of patients, even the possibility that it has of simulate psychiatric or malignant disorders like from described patients. The high clinical suspicion and appropriate diagnostic approach focused on problems, taking into account unusual entities like this, and it will make one accurate diagnostic and therapeutic approach. We hope that with this case report the medical community is encouraged to report more cases and thus in this way achieve a unanimous concept in respect to the management, diagnostic and therapy of this entity which has a high mortality rate.
